# Induction of HMOX1 by mesenchymal stem cell cytotherapy inhibits osteoclastogenesis and myeloma‐induced bone disease

**DOI:** 10.1002/ctm2.70302

**Published:** 2025-05-27

**Authors:** Xin Li, Wen Ling, Bart Barlogie, Shmuel Yaccoby

**Affiliations:** ^1^ Department of Internal Medicine Myeloma Center Winthrop P. Rockefeller Cancer Institute University ofArkansas for Medical Sciences Little Rock Arkansas USA

1

Dear Editor,

Multiple myeloma (MM) cells typically grow in focal lesions (FLs), which often turn into osteolytic lesions.^1^ Through the study of cytotherapy with mesenchymal stem cells (MSCs) for treating MM, we discovered that MSCs mediate HMOX1 expression in monocytes to balance differentiation of osteoclast precursors into osteoclasts. Lower expression of HMOX1 in the MM bone is associated with poor outcome and induction of HMOX1 pharmacologically resulted in suppression of MM‐induced bone disease.

Previously, we showed that MM‐induced osteolytic bone disease can be treated via direct cytotherapy with MSCs using our well‐established SCID‐hu and SCID‐rab MM models^2^, ^3^ (see Methods and Discussion in Supplementary Information). By applying global gene expression profiling (GEP) on the whole human bone in SCID‐hu mice we found that MSC cytotherapy induced expression of several genes associated with the macrophages and monocytes (Figure 1A, Table S1). Of the top upregulated genes, we focused on *HMOX1*, which encodes heme oxygenase 1, and known as an inducible factor that mediate oxidative stress, inflammation and bone remodelling.^4^ *HMOX1* expression in bone was consistently upregulated following MSC cytotherapy in bones engrafted with 4 different MM cell lines (Figure 1B and C). Immunohistochemistry post‐cytotherapy revealed induction of HMOX1 protein in monocytes and macrophages and some MM cells (Figure 1D). *HMOX1* expression is highest among immune cells in MM bone marrow based on publicly available scRNA‐seq data (Figure 1E–G).

**FIGURE 1:**
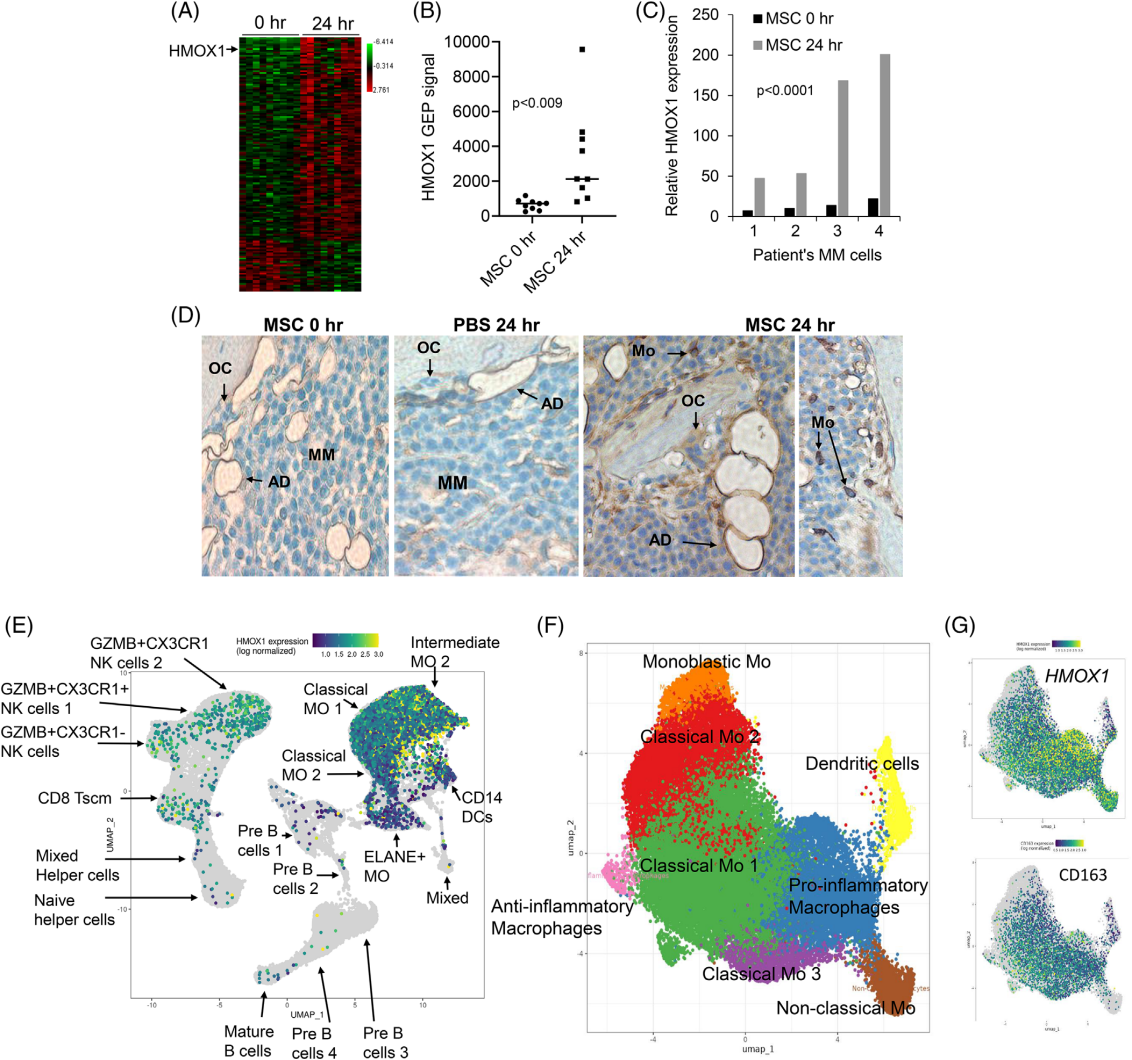
MSC cytotherapy upregulates *HMOX1* in the myelomatous human bone, a gene that is highly expressed in cells of monocytic origin. (A–D) MSCs or PBS were injected directly into the implanted human bone in MM‐bearing SCID‐hu mice. (A) A heat map of the top significantly upregulated (*n* = 122) and downregulated (*n* = 44) genes in the whole human bone engrafted with MM cells as assessed at 0 h (*n* = 9) and 24 h (*n* =  9) after injection of MSCs. (B) *HMOX1* expression in individual samples assessed via gene expression profiling. (C) *HMOX1* expression, assessed with qRT‐PCR, in myelomatous human bone engrafted with MM cells from 4 different patients. (D) Immunohistochemistry of HMOX1 in myelomatous human bones injected with MSCs or PBS at indicated timepoint (×20 original magnification). MM, multiple myeloma cells; OC, osteoclast; AD, adipocyte; Mo, monocyte. (E–G) scRNA‐seq of BM cells from healthy donors and patients with MM (see Methods in Supplementary Information). (E) Expression of *HMOX1* among various mononucleated cells. (F, G) Expression of *HMOX1* and *CD163* within subsets of monocytes/macrophages.

To study whether MSCs mediate osteoclastogenesis through HMOX1, we co‐cultured MSCs with osteoclast precursors (pOC) and found that MSCs suppressed their differentiation into multinucleated osteoclasts, an effect that was associated with upregulation of HMOX1 at the RNA and protein levels, lower expression of the osteoclast markers: *ACP5* (TRAP), *CTSK*, and *VTNR*, and lower secretion of HMGB1 (Figure 2A–G). RANKL is a master regulator of osteoclastogenesis that acts on pOC via *TNFRSF11A*/RANK. Using qRT‐PCR, immunofluorescence and immunoblot we found that MSCs conditioned medium reduced *TNFRSF11* expression and RANK levels in pOC (Figure 2H–J). MSCs secreted factors that restrain osteoclastogenesis are discussed in Supplementary Information.

**FIGURE 2:**
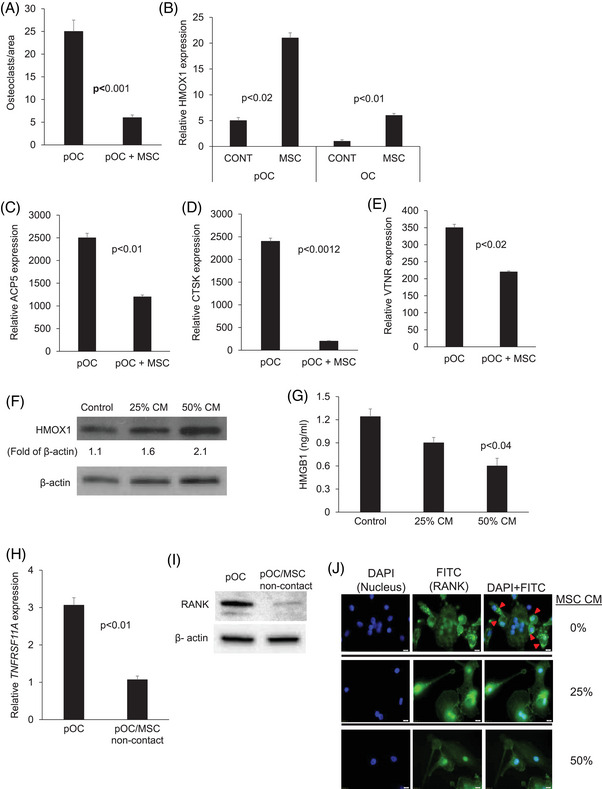
MSCs inhibit osteoclast formation, an effect associated with upregulation of *HMOX1* and reduced expression of osteoclast genes. The osteoclast precursors (pOC) were cultured alone or cocultured with MSCs in osteoclast medium. (A) Number of mature multinucleated osteoclasts on day 7. (B) *HMOX1* expression in pOC at the early phase of osteoclast differentiation (day 2) or later stage (day 7) when osteoclasts (OC) are formed. (C–E) Expression of *ACP5* (TRAP), *CTSK*, and *VTNR* on day 7. Immunoblot analysis of HMOX1 protein in pOC treated with MSC‐conditioned medium (CM) for 2 days. (F) Immunoblot for HMOX1 in pOC cultured in osteoclast medium and treated with indicated concentration of MSC‐CM for 2 days. (G) Secreted levels of HMGB1 in cultures as in F, assessed on day 7. (H–J) Osteoclast precursors were cultured alone or cocultured with MSCs in non‐contact conditions with osteoclast medium for 2 days. (H) *TNFRSF11*A expression (encoding RANK), assessed by qRT‐PCR. (I) Protein production of RANK, determined via immunoblot. (J) Immunofluorescence staining of osteoclast precursors treated with indicated per cent of MSC‐conditioned medium for 2 days. RANK is stained green with FITC‐conjugated antibody. Red arrows point to cell surface RANK.

NFκB is induced by RANK/RANKL signalling and is a vital signalling pathway for osteoclastogenesis.^5^ MSC‐conditioned medium inhibited cytoplasmic phosphorylated IκBα and NFκB p65 and nuclear NFκB p65 in pOC (Figure 3A–C).

**FIGURE 3:**
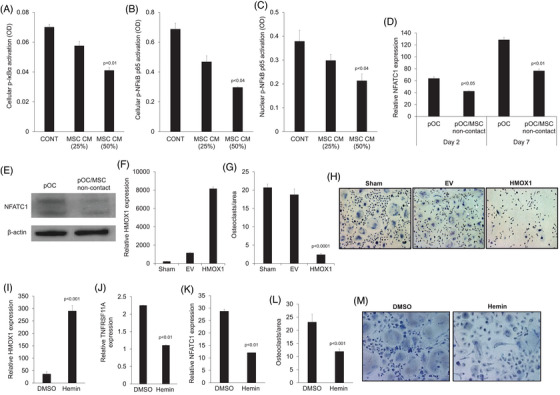
MSCs inhibit the NFκB and NFATC1 transcription factors during osteoclast differentiation while induction of HMOX1 expression inhibits osteoclastogenesis. (A–F) Osteoclast precursors (pOC) cultured in osteoclast medium and treated with fresh medium (CONT) or indicated concentrations of MSC‐conditioned medium (CM) for 2 days. (A) The effect of MSC‐CM on phosphorylation of IKBα in pOC. (B) The effect of MSC‐CM on phosphorylated NFκB p65 (p‐NFκB p65) in pOC. (C) The effect of MSC‐CM on NFκB p65 activation in the nuclear fraction of pOC. (D) *NFATC1* expression in pOC assessed with qRT‐PCR in pOC collected on day 2 and day 7. (E) Immunoblot for NFATC1 in pOC performed on day 2. (F–H) Monocytes were infected with lentivirus particles containing empty vector (EV) or *HMOX1* cDNA and cultured with osteoclast medium. (F) *HMOX1* expression 2 days after injection assessed with qRT‐PCR. (G) Number of multinucleated osteoclasts on day 7 in sham, EV, and *HOMX1* cDNA samples. (H) Representative photos of multinucleated osteoclasts of sham, EV, and *HOMX1* cDNA groups (×20 original magnification). Arrows point to multinucleated osteoclasts. (I–L) Expression of *HMOX1*, *TNFRS11A*, and *NFATC1* in osteoclast precursors treated with DMSO (control) or hemin (50 µM) for 3 days. (M) Number of TRAP+ multinucleated osteoclasts on day 7. (N) Representative TRAP staining of DMSO and hemin samples on day 7.

NFATC1 is a main downstream transcription factor activated by the RANKL/NFκB pathway in osteoclasts that induces expression of typical genes associated with osteoclasts, such as *CTSK* and *APC5/TRAP*.^6^ Compared to pOC cultured alone, pOC cultured with MSCs had lower expression of *NFATC1* (Figure 3D). Immunoblots conducted on pOC confirmed reduced levels of NFATC1 in pOC cocultured with MSCs in a non‐contact condition (Figure 3E). Taken together, these data indicate that MSCs downregulate RANK expression in pOC, resulting in reduced activation of the NFκB pathway, leading to lower activity of the main osteoclastic transcription factor, NFATC1.

We applied two different methods to shed light on the direct role of HMOX1 on osteoclast formation. To induce constitutive *HMOX1* expression, we infected monocytes with lentiviral particles containing either *HMOX1* cDNA or empty vector. Culturing these cells in osteoclast medium for 7 days induced formation of multinucleated osteoclasts in the control groups (i.e., noninfected cells [sham] and cells containing empty vector); in contrast, multinucleated osteoclasts failed to form in cells expressing *HMOX1* cDNA (Figure 3F–H).

To further corroborate our finding, we used hemin, a pharmacological agent that induces *HMOX1* expression.^7^ We confirmed by qRT‐PCR that treatment of pOC with hemin induced *HMOX1* gene expression in these cells (Figure 3I). We also tested expression of the RANKL receptor RANK, encoded by *TNFRSF11A*, and *NFATC1*. Both *TNFRSF11A* and *NFATC1* were downregulated in hemin‐treated pOC (Figure 3K–M). Treatment with hemin inhibited the formation of multinucleated osteoclasts in pOC continually cultured in osteoclast medium for 7 days (Figure 3M and N). Together, these findings indicate that induced expression of *HMOX*1 in pOC inhibits pOC differentiation into osteoclasts.

In vivo, we used our well‐established SCID‐rab model to test the effect of hemin on MM growth and MM‐induced bone disease. Specifically, we engrafted a BM‐dependent MM line into SCID‐rab mice as previously described.^8^ Upon establishment of MM engraftment, mice were treated with hemin or control vehicle (DMSO) for 4 weeks. The bone mineral density (BMD) of the implanted myelomatous bone was reduced from pretreatment levels by 16% and 1% in the DMSO‐ and hemin‐treated groups, respectively (*p*  <  .005) (Figure 4A). The X‐rays showed more osteolysis and lytic lesions in DMSO‐treated bones than in hemin‐treated bones (Figure 4B). Although fewer osteoclasts were observed in hemin‐treated bones, the number of osteoblasts were equivalent (Figure 4C and D). Further, total tumour burden analysed by circulating hIg ELISA were similar between the two groups (Figure 4E). Thus, these results indicate that hemin, the *HMOX1* inducer, inhibited osteoclastogenesis and MM‐induced osteolytic lesions in vivo.

**FIGURE 4:**
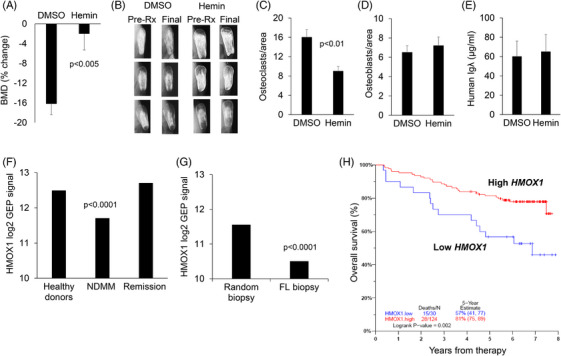
Induction of HMOX1 by hemin inhibits bone resorption in vivo while lower *HMOX1* in clinical biopsies associated with poor outcome. (A–E) MM cells were engrafted in SCID‐rab mice and upon establishment of multiple myeloma, were treated with DMSO (control, *n*  =  9) or hemin (50 µM, *n*  =  9) both diluted in 500 µL PBS and injected into the surrounding implanted bone twice a week for 4 weeks. (A) Changes in bone mineral density of the implanted bones. (B) Representative X‐ray radiographs of the implanted myelomatous bone prior to treatment (Pre‐Rx) and at the end of the experiment (Final). (C, D) Numbers of TRAP+ osteoclasts and osteocalcin+ osteoblasts. (E) Levels of human immunoglobulin lambda light chain in blood, indicative of tumour burden. (F–H) Clinical observations. (F) Expression of *HMOX1* in bone biopsies from healthy donors, newly diagnosed multiple myeloma (MM) patients (NDMM), and MM patients in remission. (G) Expression of *HMOX1* in paired biopsy samples from random BM and focal lesions (FL) of the same patients. (H) Overall survival of patients with NDMM enrolled in the TT3 clinical trial (first 8 years of trial).

To explore the clinical relevance of our findings, we used publicly available GEP data from our institute to analyse expression of *HMOX1* in whole bone biopsies from healthy donors (*n*  =  68), patients with NDMM (*n*  =  354), and MM patients in remission (*n*  =  132).^9^ Consistent with our findings, *HMOX1* expression was decreased in whole biopsies of patients with NDMM and returned to normal when patients were in remission (Figure 4F). We also used available data from paired random interstitial bone biopsy and FL biopsy from patients with NDMM (*n* = 49 patients). *HMOX1* expression was lower in FLs than in interstitial bone samples (Figure 4G). Additionally, lower expression of *HMOX1* was associated with poor overall survival in patients with NDMM enrolled in a TT3 clinical trial at University of Arkansas for Medical Sciences (Figure 4H).^10^ These observations indicate that lower *HMOX1* expression in myelomatous bones is markedly suppressed in FLs and that lower expression in interstitial bone marrow is an adverse clinical parameter.

We conclude that MSCs are central in mediating differentiation of osteoclasts through maintaining high expression of HMOX1 in monocytes. Suppression of bone resorption by MSC cytotherapy is partially mediated by induction of HMOX1 in monocytes suggesting that approaches to induce *HMOX1* expression may help control MM‐induced osteolysis.

## AUTHOR CONTRIBUTIONS

X.L. performed the in vitro and in vivo work, the GEP analysis, immunohistochemistry, immunoblots, qRT‐PCR, and statistical analyses; X.L. was also one of the writers of the paper. W.L. performed in vitro and in vivo work and the immunohistochemistry. B.B. interpreted the data and provided clinical insight. S.Y. designed and directed the research, conceptualised the work, analysed and interpreted the data, and was one of the writers of the paper.

## CONFLICT OF INTEREST STATEMENT

The authors declare no competing financial interests.

## FUNDING

This work was supported by a grant CA55819 (B.B.) from the National Cancer Institute and grant CA200068 (S.Y.) from the US Department of Defense.

## ETHICS STATEMENT

All animal experimental procedures and protocols were approved by the University of Arkansas for Medical Sciences Institutional Animal Care and Use Committee.

## Supporting information



Supporting information

Supporting information

## Data Availability

The GEP analyses of bone biopsies from MM patients and healthy donors are available as described by Danziger et al. (2020).^9^

## References

[1] Yaccoby S . Advances in the understanding of myeloma bone disease and tumour growth. Br J Haematol. 2010;149(3):311‐321. doi:10.1111/j.1365-2141.2010.08141.x 20230410 PMC2864366

[2] Li X , Ling W , Pennisi A , et al. Human placenta‐derived adherent cells prevent bone loss, stimulate bone formation, and suppress growth of multiple myeloma in bone. Stem Cells. 2011;29(2):263‐273. doi:10.1002/stem.572 21732484 PMC3175303

[3] Li X , Ling W , Khan S , Yaccoby S . Therapeutic effects of intrabone and systemic mesenchymal stem cell cytotherapy on myeloma bone disease and tumor growth. J Bone Miner Res. 2012;27(8):1635‐1648. doi:10.1002/jbmr.1620 22460389 PMC3395777

[4] Zhou X , Yuan W , Xiong X , et al. HO‐1 in bone biology: potential therapeutic strategies for osteoporosis. Front Cell Dev Biol. 2021;9:791585. doi:10.3389/fcell.2021.791585 34917622 PMC8669958

[5] Boyce BF , Li J , Yao Z , Xing L . Nuclear factor‐kappa B regulation of osteoclastogenesis and osteoblastogenesis. Endocrinol Metab (Seoul). 2023;38(5):504‐521. doi:10.3803/EnM.2023.501 37749800 PMC10613774

[6] Omata Y , Tachibana H , Aizaki Y , Mimura T , Sato K . Essentiality of Nfatc1 short isoform in osteoclast differentiation and its self‐regulation. Sci Rep. 2023;13(1):18797. doi:10.1038/s41598-023-45909-3 37914750 PMC10620225

[7] Sakai E , Shimada‐Sugawara M , Nishishita K , et al. Suppression of RANKL‐dependent heme oxygenase‐1 is required for high mobility group box 1 release and osteoclastogenesis. J Cell Biochem. 2012;113(2):486‐498. doi:10.1002/jcb.23372 21928347

[8] Li X , Pennisi A , Zhan F , Sawyer JR , Shaughnessy JD , Yaccoby S . Establishment and exploitation of hyperdiploid and non‐hyperdiploid human myeloma cell lines. Br J Haematol. 2007;138(6):802‐811. doi:10.1111/j.1365-2141.2007.06742.x 17760811 PMC2748973

[9] Danziger SA , McConnell M , Gockley J , et al. Bone marrow microenvironments that contribute to patient outcomes in newly diagnosed multiple myeloma: a cohort study of patients in the Total Therapy clinical trials. PLoS Med. 2020;17(11):e1003323. doi:10.1371/journal.pmed.1003323 33147277 PMC7641353

[10] Barlogie B , Mitchell A , van Rhee F , Epstein J , Morgan GJ , Crowley J . Curing myeloma at last: defining criteria and providing the evidence. Blood. 2014;124(20):3043‐3051. doi:10.1182/blood-2014-07-552059 25293776 PMC4231416

