# Supplementary material for: Induction of HMOX1 by mesenchymal stem cell cytotherapy inhibits osteoclastogenesis and myeloma‐induced bone disease
Source: Clin Transl Med. 2025 May 27;15(5):e70302. doi: 10.1002/ctm2.70302 (PMC12116329; doi:10.1002/ctm2.70302)
Supplement: Supplementary file 2 — Supporting information [file CTM2-15-e70302-s001.docx]

**Supplementary Information**

**METHODS**

**In Vivo Studies**

The SCID-hu and SCID-rab mouse models for MM were constructed as previously described.^1,2^ The growth of MM and the induction of bone disease are comparable between these two experimental models. The SCID-hu model allowed the assessment of human gene expression in a system where the MM cells, MSCs, and bone are all human origin. The SCID-rab model facilitated the use of a large number of mice per group to evaluate the treatment effects of the HMOX1 inducer, hemin, on MM-induced bone disease. A BM-dependent MM cell line was used for engraftment of MM in SCID-hu or SCID-rab mice as previously described.^3,4^ All experimental procedures and protocols were approved by the University of Arkansas for Medical Sciences Institutional Animal Care and Use Committee.

For MSC cytotherapy*,* cultures of normal MSCs were prepared as described.^5-9^ MSC cytotherapy within the implanted myelomatous bone was performed as previously described.^7,8^ For the GEP study and immunohistochemistry analyses, normal MSCs (1x10^6^ cells/bone) were injected into the implanted human bone in MM-bearing SCID-hu mice and analyzed immediately (0 hours) and at 24 hours. As an additional control group, the implanted bones were injected with PBS and analyzed after 24 hours.

For hemin treatment, MM-bearing SCID-rab mice were subcutaneously treated with DMSO (control, n = 9) or hemin (50 µM, n = 9), both diluted in 500 µl PBS and injected into the surrounding implanted bone, twice a week for 4 weeks. Mice were periodically bled from the tail vein, and changes in levels of circulating human immunoglobulins (hIg, indicator of MM burden) were determined by enzyme-linked immunosorbent assay (ELISA).^1,2^ Radiographs were taken with an AXR Minishot-100 beryllium source instrument (Associated X-Ray Imaging Corp., Haverhill, MA, USA). Changes in bone mineral density (BMD) of the implanted bone were determined with a PIXImus DEXA (GE Medical Systems LUNAR, Madison, WI, USA).^4,10^

**Global Gene Expression Profiling**

GEP was performed with the Affymetrix U133-Plus microarray, which contains approximately 54,000 genes (Affymetrix, Santa Clara, CA, USA) as previously described.^11^ The biopsy samples used to generate the GEP data are available at GEO DataSets (GSE136324 and GSE136337), and data sets are available and described elsewhere.^12^

For the animal model study, myelomatous implanted fetal human bones (femur or tibia) were removed from SCID-hu mice at 0 hours (n = 9) or 24 hours (n = 9) after the injection of MSCs and were used for RNA extraction and GEP analyses.

For clinical samples, preparation and RNA extraction from bone biopsies from individual healthy donors and from patients with MM was previously described in detail.^12,13^ GEP data were available for baseline BM biopsy samples from healthy donors (n = 68), patients with newly diagnosed MM (NDMM, n = 354), or MM patients in remission (n = 132).^12,13^ Also, paired biopsy samples (i.e., from the same MM patient) of interstitial random BM of the iliac crest or from CT-guided fine-needle biopsies of MRI-defined FLs (n = 49) were available.

**Quantitative Real-Time Polymerase Chain Reaction (qRT-PCR)**

Total RNA (1 µg) from each sample was reverse-transcribed with the SuperScript III First-Strand Synthesis SuperMix for qRT-PCR (Invitrogen Corp., Carlsbad, CA, USA). The quantitative real-time polymerase chain reaction (qRT-PCR) was performed with the TaqMan gene expression assay on an ABI Prism 7000 sequence analyzer according to the manufacturer’s recommended protocol (Applied Biosystems, Foster City, CA, USA). Reverse-transcribed RNA (10 ng) was amplified with the TaqMan Universal PCR Master Mix and TaqMan gene expression assays (ID HS99999905_m1 for GAPDH as an endogenous control, ID Hs0110250_m1 for HMOX1, ID Hs00166156_m1 for CTSK, ID Hs00187192_m1 for RANK, ID Hs00542678_m1 for NFATC1, ID Hs00356261_m1 for TRAP, ID Hs00233808_m1 for VTNR). Each reaction was run in duplicate. The comparative threshold cycle method was used to calculate the amplification fold, as specified by the manufacturer.

**Immunohistochemistry and Histochemistry**

Immunohistochemistry for osteocalcin was performed to identify osteoblasts, and tartrate-resistant acid phosphatase (TRAP) staining was used to detect osteoclasts. The number of osteoclasts and the number of osteoblasts were quantified as previously described.^4^ A ZEISS AXIO Observer.A1 microscope (Delta Optical Instruments, North Little Rock, AR, USA) was used to obtain images with a SPOT 2 digital camera (Diagnostic Instruments Inc., Sterling Heights, MI, USA).

The Intracellular Immunofluorescence Cell Staining (labeling) Kit (IFN-1, FIVEphoton Biochemicals, San Diego, CA, USA) was used for RANK immunofluorescence staining. In this experiment, goat anti-rabbit IgG-FITC (cs-2012, Santa Cruiz Biotechnology, Inc., Dallas, TX, USA), RANK antibody (H-300, Santa Cruiz Biotechnology, Inc., Dallas, TX, USA), and goat serum (sc-2043, Santa Cruiz Biotechnology, Inc., Dallas, TX, USA) were used as recommended by the manufacturer. Procedures followed the protocol provided in the cell staining kit. Briefly, osteoclast cells were cultured on a small cover glass. Cells for staining were fixed with methanol and air-dried. Cells were then washed with PBS, permeabilized, and blocked for 15 minutes before addition of primary antibody diluted in block. Cells were left in primary antibody for 1 hour. Cover slips were then rinsed with reagents provided in the kit before the secondary antibody was applied for 1 hour in the dark. After 1 hour, slides were washed with reagents provided in the kit. Cells were then counterstained with DAPI, and slides were mounted and examined under the light microscope (Olympus BX60). Cell photos were taken by digital camera (Olympus XM-10), and images were processed with the Olympus CellSens software.

**Immunoblotting**

The antibodies for HMOX1 (LS-B3692), NFATC1 (LS-B1977), and β-actin (LS-B4080) were from LifeSpan BioSciences, Seattle, WA, USA; anti-RANK (AB1861) was from Millipore, Billerica, MA, USA.

The Nuclear/Cytosol Fractionation Kit (BioVision, Mountain View, CA, USA) was used to isolate cytosolic and nuclear fractions of osteoclast precursors. Equal amounts of lysate were separated by electrophoresis on 4% to 12% sodium dodecyl sulfate-polyacrylamide gels, and immunoblotting was carried out according to the Western Breeze chemiluminescent immunodetection protocol as described by the manufacturer (Invitrogen).

**Enzyme-Linked Immunosorbent Assay**

NFκB activation and HMGB1 were analyzed via ELISA. InstantOne ELISA for NFκB pathway activation (Ref 85-86084-11) was purchased from eBioscience, Inc., San Diego, CA, USA. HMGB1 ELISA kit (EB0H0009H) was purchased from NeoBioLab, NEO Group Inc., Cambridge, MA, USA. ELISAs were performed according to protocols provided in the kit.

**Preparation of Osteoclasts**

Human pOC were prepared as previously described.^14^ Briefly, blood mononuclear cells were cultured in 24-well plates at 2.5×10^6^ cells/mL in an osteoclast medium: minimal essential medium supplemented with 10% fetal bovine serum, receptor activator for NFκB ligand (RANKL, 50 ng/mL, PeproTech Inc., Rocky Hill, NJ, USA), M-CSF (25 ng/mL, PeproTech Inc., Rocky Hill, NJ, USA), and an antibiotic mixture (penicillin, streptomycin, and neomycin; Gibco, Grand Island, NY, USA). Cells were cultured for 3 to 4 days, at which time the nonadherent cells were removed; the remaining adherent cells were used as pOC. Note that osteoclast medium was used for the entire study.

For coculture experiments, MSCs were cultured in the upper chamber of 1 µm trans-well inserts (non-contact conditions) and placed in 24-well plates containing pOC in the bottoms of the wells. pOC were cultured in the absence or presence of MSC conditioned medium (50%) or cocultured with MSCs in non-contact conditions for 6 days and then subjected to TRAP staining with the use of an acid phosphatase kit (Sigma).

**Statistical Analysis**

All values are expressed as mean ± SEM. The in vitro assays and the effect of treatment on BMD, MM tumor burden, osteoblast and osteoclast numbers, and GEP were analyzed with the Student’s t-test. R-package “SAM” was used to assess the top upregulated and downregulated genes following MSC cytotherapy. A difference in expression of a probe set was identified as significant if (**a**) the comparison between the two groups had *P* < 0.05 with Student's *t*-test, (**b**) the mean signal was > 300 in the 24-hour MSC group when assessing upregulated genes and > 300 in the 0-hour control group when assessing downregulated genes, and (**c**) the comparison had an absolute fold change > 2. These genes were applied for preparation of the heatmap.

**Data Availability**

The scRNA-Seq data of immune cells in BM of patients with MM and healthy donors are available as described in De Jong at el., 2021 and 2024.^15,16^ The GEP analyses of bone biopsies from MM patients and healthy donors are available as described by Danziger et al., 2020.^12^

**DISCUSSION**

Our study provides some understanding of the mechanism by which MSC cytotherapy prevents MM-induced bone disease and provides insight into the interaction of MSCs with pOC. We found that factors secreted by MSCs resulted in an immediate downregulation of RANK and upregulation of HMOX1 in pOC, preventing these cells from differentiating into mature osteoclasts when exposed to RANKL. Key downstream mediators secreted by pOC—including NFkB, the master osteoclastogenic transcription factor NFATC1, and HMGB1—were inhibited under the following conditions: upregulation of *HMOX1* by MSCs; induction of *HMOX1* via lentiviral expression; and treatment with hemin, a pharmacological inducer of *HMOX1*. In vivo, induction of HMOX1 with hemin prevented MM-induced bone disease through reduced osteoclastogenesis. Using publicly available global GEP and scRNA-seq data of clinical samples, we found that monocytes are the main cell types that express *HMOX1* in the immune BM and that *HMOX1* expression in whole bone biopsies taken from FLs is lower than its expression in interstitial BM from the same patients with MM. Finally, lower levels of *HMOX1* in random bone biopsies is associated with shorter overall survival. These finding indicate a crucial role for *HMOX1* in controlling MM bone disease and in inducing a microenvironment that favors survival of patients with MM.

The heme oxygenase system, including heme-binding proteins, heme transporters, and the heme degrading protein heme oxygenase, are known to increase cellular antioxidant and anti-inflammatory properties through a battery of cytoprotective systems. The heme oxygenase gene has two distinct isoforms, *HMOX1* and *HMOX2*, both of which have similar enzymatic activity and are the rate-limiting microsomal enzyme which degrades heme to carbon monoxide, ferrous iron, and biliverdin. *HMOX2* is constitutively expressed and present at high concentrations in the brain and testes, whereas *HMOX1* is ubiquitously distributed and strongly induced by oxidative nitrosative, osmotic, and hemodynamic stresses.^17^ HMOX1 and its enzymatic products are anti-inflammatory, cytoprotective, and protect the vasculature.^18^ Thus, unlike HMOX2, the inducible HMOX1 plays a crucial role in mediating stress responses. Its reduced expression in the bones of MM patients offers significant insights into how MM suppresses the BM microenvironment, leading to manifestations such as FLs. Although studies have documented the ability of HMOX1 to inhibit osteoclast differentiation,^18,19^ to our knowledge, the link between MSC cytotherapy and induction of *HMOX1* expression in cells of monocytic lineage has not been previously reported and contributes to our understanding of the interaction between MSCs and osteoclast precursors. Although other cells in the BM, such as adipocytes and MM cells, may be induced to express *HMOX1*, as shown in Figure 1D, the scRNA-seq atlas by De Jong et al., 2021,^15^ also analyzed MM cells and demonstrated low expression of HMOX1 in these cells. Gene expression of mature adipocytes is difficult to analyze at the single-cell level due to their fragility.

Our finding that macrophage-associated genes are among the top genes upregulated in bone following MSC cytotherapy further supports the role of macrophages in regenerative medicine, as macrophages reportedly mediate tissue repair.^20^ Recent studies indicated that monocytes and macrophages are significantly depleted in MM FLs compared with interstitial BM^21^ and that a higher proportion of these cells in interstitial BM is associated with favorable outcomes for patients with MM who receive immune-based therapy.^22^ We previously analyzed MSC gene expression in bone biopsies from MM patients and established a 3-gene score that is associated with patients’ outcome.^13^ We also found that higher MSC gene-score is associated with suppression of immune cells, including monocytes and macrophages.^13^ These findings, in combination with our current data, suggest that the altered composition and function of MSCs contribute to downregulation of *HMOX1*, thus forcing monocytes to differentiate into pOC, which are abundant along the bone surface in MM osteolytic lesions. Our data on the therapeutic potential of MSCs via HMOX1 induction align with the phenotype of HMOX1-overexpressing macrophages in various contexts, demonstrating anti-inflammatory properties, enhanced redox homeostasis, metabolic adaptation, and inhibition of osteoclastogenesis.^18,23^

While our previous^7,8^ and current in vivo studies indicate the therapeutic potential of direct MSC cytotherapy in MM, our findings with the use of the MSC secretome also suggest that increased activity of endogenous MSCs is beneficial for the MM BM. Regarding cytotherapy, MSCs have demonstrated therapeutic effects in various contexts and have not shown any risk of tumorigenicity.^24^ This may be due to the fact that these cells are normal and do not acquire genetic alterations. The cytotherapeutic cells are also known to exert immediate effects on the surrounding microenvironment and often disappear shortly after injection. Our current study, showing the recruitment of cells such as HMOX1-expressing monocytes, provides insight into the mechanisms by which MSC cytotherapy exerts bone healing effects. We have previously shown that MSCs express several secreted factors that could inhibit osteoclast differentiation,^8,13^ including *CYR61*/CCN1,^25^ decorin^26^ and thrombospondin-1.^27^

In summary, through the study of MSC cytotherapy, we discovered that MSCs mediate HMOX1 expression in monocytes to balance differentiation of osteoclast precursors into osteoclasts. Approaches to induce *HMOX1* expression may help control MM-induced osteolysis.

**REFERENCES**

1. Yaccoby S, Barlogie B, Epstein J. Primary myeloma cells growing in SCID-hu mice: a model for studying the biology and treatment of myeloma and its manifestations. *Blood*. Oct 15 1998;92(8):2908-13.

2. Yata K, Yaccoby S. The SCID-rab model: a novel in vivo system for primary human myeloma demonstrating growth of CD138-expressing malignant cells. *Leukemia*. Nov 2004;18(11):1891-7. doi:10.1038/sj.leu.2403513

3. Li X, Pennisi A, Zhan F, Sawyer JR, Shaughnessy JD, Yaccoby S. Establishment and exploitation of hyperdiploid and non-hyperdiploid human myeloma cell lines. *Br J Haematol*. Sep 2007;138(6):802-11. doi:10.1111/j.1365-2141.2007.06742.x

4. Pennisi A, Ling W, Li X, et al. Consequences of daily administered parathyroid hormone on myeloma growth, bone disease, and molecular profiling of whole myelomatous bone. *PLoS One*. Dec 20 2010;5(12):e15233. doi:10.1371/journal.pone.0015233

5. Yaccoby S, Wezeman MJ, Zangari M, et al. Inhibitory effects of osteoblasts and increased bone formation on myeloma in novel culture systems and a myelomatous mouse model. *Haematologica*. Feb 2006;91(2):192-9.

6. Pennisi A, Ling W, Li X, et al. The ephrinB2/EphB4 axis is dysregulated in osteoprogenitors from myeloma patients and its activation affects myeloma bone disease and tumor growth. *Blood*. Aug 27 2009;114(9):1803-12. doi:10.1182/blood-2009-01-201954

7. Li X, Ling W, Pennisi A, et al. Human placenta-derived adherent cells prevent bone loss, stimulate bone formation, and suppress growth of multiple myeloma in bone. *Stem Cells*. Feb 2011;29(2):263-73. doi:10.1002/stem.572

8. Li X, Ling W, Khan S, Yaccoby S. Therapeutic effects of intrabone and systemic mesenchymal stem cell cytotherapy on myeloma bone disease and tumor growth. *J Bone Miner Res*. Aug 2012;27(8):1635-48. doi:10.1002/jbmr.1620

9. Mehdi SJ, Johnson SK, Epstein J, et al. Mesenchymal stem cells gene signature in high-risk myeloma bone marrow linked to suppression of distinct IGFBP2-expressing small adipocytes. *Br J Haematol*. Feb 2019;184(4):578-593. doi:10.1111/bjh.15669

10. Pennisi A, Li X, Ling W, Khan S, Zangari M, Yaccoby S. The proteasome inhibitor, bortezomib suppresses primary myeloma and stimulates bone formation in myelomatous and nonmyelomatous bones in vivo. *Am J Hematol*. Jan 2009;84(1):6-14. doi:10.1002/ajh.21310

11. Zhan F, Huang Y, Colla S, et al. The molecular classification of multiple myeloma. *Blood*. Sep 15 2006;108(6):2020-8. doi:10.1182/blood-2005-11-013458

12. Danziger SA, McConnell M, Gockley J, et al. Bone marrow microenvironments that contribute to patient outcomes in newly diagnosed multiple myeloma: A cohort study of patients in the Total Therapy clinical trials. *PLoS Med*. Nov 2020;17(11):e1003323. doi:10.1371/journal.pmed.1003323

13. Schinke C, Qu P, Mehdi SJ, et al. The Pattern of Mesenchymal Stem Cell Expression Is an Independent Marker of Outcome in Multiple Myeloma. *Clin Cancer Res*. Jun 15 2018;24(12):2913-2919. doi:10.1158/1078-0432.CCR-17-2627

14. Yaccoby S, Wezeman MJ, Henderson A, et al. Cancer and the microenvironment: myeloma-osteoclast interactions as a model. *Cancer Res*. Mar 15 2004;64(6):2016-23. doi:10.1158/0008-5472.can-03-1131

15. de Jong MME, Kellermayer Z, Papazian N, et al. The multiple myeloma microenvironment is defined by an inflammatory stromal cell landscape. *Nat Immunol*. Jun 2021;22(6):769-780. doi:10.1038/s41590-021-00931-3

16. de Jong MME, Fokkema C, Papazian N, et al. An IL-1beta-driven neutrophil-stromal cell axis fosters a BAFF-rich protumor microenvironment in individuals with multiple myeloma. *Nat Immunol*. May 2024;25(5):820-833. doi:10.1038/s41590-024-01808-x

17. Maines MD. The heme oxygenase system: a regulator of second messenger gases. *Annu Rev Pharmacol Toxicol*. 1997;37:517-54. doi:10.1146/annurev.pharmtox.37.1.517

18. Zhou X, Yuan W, Xiong X, et al. HO-1 in Bone Biology: Potential Therapeutic Strategies for Osteoporosis. *Front Cell Dev Biol*. 2021;9:791585. doi:10.3389/fcell.2021.791585

19. Sakai E, Shimada-Sugawara M, Nishishita K, et al. Suppression of RANKL-dependent heme oxygenase-1 is required for high mobility group box 1 release and osteoclastogenesis. *J Cell Biochem*. Feb 2012;113(2):486-98. doi:10.1002/jcb.23372

20. Yu Y, Yue Z, Xu M, et al. Macrophages play a key role in tissue repair and regeneration. *PeerJ*. 2022;10:e14053. doi:10.7717/peerj.14053

21. John L, Poos AM, Brobeil A, et al. Resolving the spatial architecture of myeloma and its microenvironment at the single-cell level. *Nat Commun*. Aug 17 2023;14(1):5011. doi:10.1038/s41467-023-40584-4

22. Maura F, Boyle EM, Coffey D, et al. Genomic and immune signatures predict clinical outcome in newly diagnosed multiple myeloma treated with immunotherapy regimens. *Nat Cancer*. Dec 2023;4(12):1660-1674. doi:10.1038/s43018-023-00657-1

23. Yeudall S, Upchurch CM, Leitinger N. The clinical relevance of heme detoxification by the macrophage heme oxygenase system. *Front Immunol*. 2024;15:1379967. doi:10.3389/fimmu.2024.1379967

24. Margiana R, Markov A, Zekiy AO, et al. Clinical application of mesenchymal stem cell in regenerative medicine: a narrative review. *Stem Cell Res Ther*. Jul 28 2022;13(1):366. doi:10.1186/s13287-022-03054-0

25. Johnson SK, Stewart JP, Bam R, et al. CYR61/CCN1 overexpression in the myeloma microenvironment is associated with superior survival and reduced bone disease. *Blood*. Sep 25 2014;124(13):2051-60. doi:10.1182/blood-2014-02-555813

26. Li X, Pennisi A, Yaccoby S. Role of decorin in the antimyeloma effects of osteoblasts. *Blood*. Jul 1 2008;112(1):159-68. doi:10.1182/blood-2007-11-124164

27. Amend SR, Uluckan O, Hurchla M, et al. Thrombospondin-1 regulates bone homeostasis through effects on bone matrix integrity and nitric oxide signaling in osteoclasts. *J Bone Miner Res*. Jan 2015;30(1):106-15. doi:10.1002/jbmr.2308
